# Regenerative Response of Degenerate Human Nucleus Pulposus Cells to GDF6 Stimulation

**DOI:** 10.3390/ijms21197143

**Published:** 2020-09-27

**Authors:** Tom Hodgkinson, Hamish T. J. Gilbert, Tej Pandya, Ashish D. Diwan, Judith A. Hoyland, Stephen M. Richardson

**Affiliations:** 1 Division of Cell Matrix Biology and Regenerative Medicine, School of Biological Sciences, Faculty of Biology, Medicine and Health, University of Manchester, Manchester Academic Health Sciences Centre, Oxford Road, Manchester M13 9PT, UK; tomhodgkinson@rcsi.ie (T.H.); hamish.gilbert@manchester.ac.uk (H.T.J.G.); tej.pandya@nhs.net (T.P.); judith.hoyland@manchester.ac.uk (J.A.H.); 2 St George & Sutherland Clinical School, University of New South Wales, Sydney, NSW 2217, Australia; a.diwan@unsw.edu.au; 3 NIHR Manchester Biomedical Research Centre, Central Manchester Foundation Trust, Manchester Academic Health Science Centre, Manchester M13 9NT, UK

**Keywords:** intervertebral disc degeneration, growth differentiation factor 6, nucleus pulposus, regenerative medicine, growth factor signalling

## Abstract

Growth differentiation factor (GDF) family members have been implicated in the development and maintenance of healthy nucleus pulposus (NP) tissue, making them promising therapeutic candidates for treatment of intervertebral disc (IVD) degeneration and associated back pain. GDF6 has been shown to promote discogenic differentiation of mesenchymal stem cells, but its effect on NP cells remains largely unknown. Our aim was to investigate GDF6 signalling in adult human NP cells derived from degenerate tissue and determine the signal transduction pathways critical for GDF6-mediated phenotypic changes and tissue homeostatic mechanisms. This study demonstrates maintained expression of GDF6 receptors in human NP and annulus fibrosus (AF) cells across a range of degeneration grades at gene and protein level. We observed an anabolic response in NP cells treated with recombinant GDF6 (increased expression of matrix and NP-phenotypic markers; increased glycosaminoglycan production; no change in catabolic enzyme expression), and identified the signalling pathways involved in these responses (SMAD1/5/8 and ERK1/2 phosphorylation, validated by blocking studies). These findings suggest that GDF6 promotes a healthy disc tissue phenotype in degenerate NP cells through SMAD-dependent and -independent (ERK1/2) mechanisms, which is important for development of GDF6 therapeutic strategies for treatment of degenerate discs.

## 1. Introduction

The pathogenesis and progression of intervertebral disc (IVD) degeneration is associated with phenotypic changes to cells of both the annulus fibrosus (AF) and, more notably, the central nucleus pulposus (NP) region of the IVD, ultimately compromising tissue function and integrity. Degenerate NP cells have a catabolic phenotype characterised by increased matrix-degrading enzyme production and altered extracellular matrix (ECM) synthesis, which leads to overall matrix degradation and secretion of a more fibrous ECM, along with neovascularisation and nerve ingrowth [1,2,3]. Numerous regenerative strategies designed to reverse this process and promote anabolic healthy NP gene and protein expression through targeted delivery of growth factors have been tested in vitro, including transforming growth factor (TGFβ), epidermal growth factor (EGF), fibroblast growth factor (FGF) and insulin-like growth factor 1 (IGF-1) [4,5,6,7,8]. However, use of these factors is linked with concerns that their use may cause unwanted nerve and blood vessel ingrowth, accelerating IVD degeneration. Therefore, members of the growth differentiation factor (GDF) family, the receptors for which are not expressed by invading blood vessels within degenerate discs [9], have emerged as promising candidates [10]. However, what remains unknown is whether degenerate cells can respond to GDF stimuli in a manner that reverses the cascade of degeneration affecting the disc as an organ.

The GDF family, containing GDF5, 6 and 7, is a subfamily of the bone morphogenetic protein (BMP) family. GDF family members, in particular GDF5 and GDF6, demonstrate significant and overlapping roles in the development of cartilaginous tissues, intervertebral discs and joints. GDF5/6-knockout mice display scoliosis and an altered IVD ECM composition similar to that of degenerated IVDs, i.e., a dense type I collagen matrix lacking in proteoglycan and type II collagen [11,12]. GDF6 has also been identified in the developing [13] and adult human spine [14], and mutations in the GDF6 gene are associated with defective vertebral segmentation in Klippel-Feil syndrome, a human condition where discs in the cervical region do not develop, leading to cervical vertebrae fusion [15]. In vitro GDF6 upregulates NP-marker gene expression (COL2, AGC and CKT8, 18 and 19), and proteoglycan production in human mesenchymal stem cells [16] and inhibits expression of both early and late markers of osteogenic differentiation [17]. NP cells cultured in alginate beads in vitro increased proteoglycan production with GDF6 stimulation [18], while in vivo GDF6 arrested the progression of IVD degeneration in an ovine annular tear model [19]. GDF6 injection has recently been shown to reduce pro-inflammatory and pain-related factors in a rat model of IVD degeneration, with a reduction in both mechanical and thermal-activated pain nociception [20]. This evidence suggests a definite role for GDF6 as a promising biologic in IVD regeneration and pain alleviation with a reduced risk of ectopic bone formation. However, details on the effects and mechanism of action of GDF6 signalling in human NP and AF cells are yet to be fully understood. In order to develop effective GDF6 therapies for regeneration of the IVD, it is important to fully understand its role and associated signalling pathways in healthy and degenerate human IVD biology.

GDF5, 6 and 7 (BMP 14, 13 and 12, respectively) differ from other BMP members in that they promote cartilaginous and tendon phenotypes but are incapable of stimulating bone induction [21]. GDF family proteins, similarly to other BMPs, signal through heteromeric transmembrane serine-threonine kinase receptor complex types I and II [22,23]. GDF5 and 6 signal through either of the type I receptors, BMP receptor type 1A (BMPR1A) or BMPR1B, and one of the type II receptors, BMP receptor II (BMPR2), activin A receptor type IIA (ACVR2A) or ACVR2B [24,25,26]. Downstream of GDF protein–receptor binding, SMAD1/5/8 becomes phosphorylated, resulting in transcription of lineage-specific transcription factors (e.g., SRY-box transcription factor 9 (SOX9)). GDF proteins can also activate non-SMAD pathways, including ERK1/2 (extracellular-related signal kinase) and p38 MAPK (mitogen-activated protein kinase) signalling hubs [27,28,29]. These kinase signalling hubs also modulate proinflammatory signalling [30,31], which is often raised in degenerate NP cells [32], suggesting a point at which cross-talk between anabolic and catabolic signalling may occur. Unravelling the signalling networks operating in NP cells treated with GDF6 will be important in order to develop and modulate a successful regenerative therapy for repair of degenerate discs.

The aims of this study were to investigate the expression of GDF6 receptors in NP and AF cells derived from degenerate human IVDs, and to evaluate the effects of recombinant human (rh)GDF6 as a regenerative molecule (biologic) to promote anabolic phenotypes in degenerate NP cells. The effect of 100 ng mL^−1^ rhGDF6 treatment on NP marker gene expression and sulphated glycosaminoglycan (sGAG) production was assessed, and the involvement of SMAD and non-SMAD intracellular signalling pathways investigated using inhibitors for SMAD, ERK and MAPK.

## 2. Results

### 2.1. GDF6 Receptor Expression in NP and AF Cells

**Figure 1:**
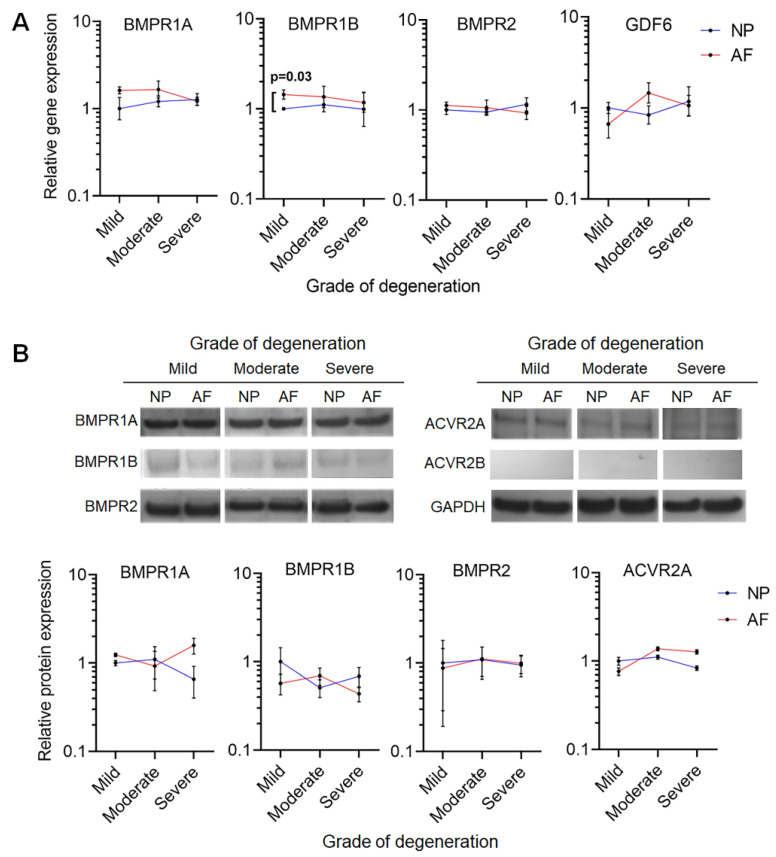
(**A**) Gene expression of GDF6 and associated receptors in nucleus pulposus (NP) and annulus fibrosus (AF) cells of different grades of degeneration relative to MRPL19 and normalised to NP cells of mild degeneration grade. Mild degeneration included grades 3–6, moderate degeneration included grades 7–9, and severe degeneration included grades 10–12, using our in-house grading scheme [33]. Gene expression is plotted as log10 of 2-ddCt, (*n* = 24). Data are means ± SEM. Statistical significance was determined by Mann–Whitney U test between cell types at the same grading, and Brown–Forsythe and Welch ANOVA test for multiple testing between grades. (**B**) Western blot images and densitometric analysis comparing bone morphogenetic protein (BMP) receptor expression in NP and AF cells isolated from degenerate intervertebral discs (IVDs) (*n* = 9 donor-matched NP and AF cells). Consistent receptor expression was observed between NP and AF tissue and between donors. Data are means ± SEM. Statistical significance was determined by Mann–Whitney tests.

The expression of key GDF6 receptors in NP and AF cells derived from mild, moderate and severely degenerated IVDs was assessed using quantitative real-time PCR (Figure 1A) and Western blot (Figure 1B). Type I receptors BMPR1A and BMPR1B along with type II receptor BMPR2 and the growth factor GDF6 were expressed at the gene level in both NP and AF cells, with grade of degeneration having no significant effect on the level of expression. All assessed genes were expressed at similar levels between NP and AF cells, with the exception of BMPR1B at mild grade which was slightly elevated (x1.4, *p* = 0.03) in AF cells compared to NP (Figure 1A; Supplementary Figure S1). The expression of type I (BMPR1A and BMPR1B) and II receptors (BMPR2 and ACVR2A) at the protein level was maintained across all grades of degeneration in both NP and AF cells (Figure 1B). We were unable to detect any protein for the type II receptor ACVR2B (Figure 1B). There was no significant difference in the level of protein expression for any of the receptors assessed across any of the grades of degeneration, or between cell types (Figure 1B). The presence of GDF6 receptors in the NP and AF cells of severely degenerative IVDs suggested that degenerate cells maintained the necessary receptors to respond to GDF6 signalling.

### 2.2. GDF6 Stimulation Upregulates the Expression of Healthy NP Marker Genes in NP Cells in Culture

**Figure 2:**
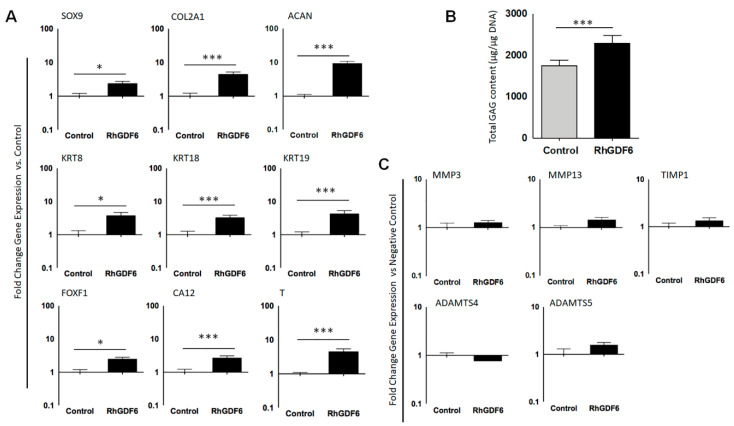
(**A**) Quantitative real-time polymerase chain reaction (QRT-PCR) analysis of healthy NP marker gene expression in NP cells with and without rhGDF6 stimulation (*n* = 18; * *p* < 0.05, *** *p* < 0.0001). Data are means *±* SEM. Statistical significance was determined by unpaired T-tests with Welch’s correction. (**B**) DMMB analysis of sGAG production in NP cells with and without rhGDF6 stimulation. Data are means *±* SEM (*n* = 18). Statistical significance was determined by unpaired T-tests with Welch’s correction. (**C**) QRT-PCR analysis of matrix remodelling enzyme gene expression in NP cells with and without rhGDF6 stimulation. Data are means *±* SEM (*n* = 18). Statistical significance was determined by Mann–Whitney tests.

To determine the effects of GDF6 stimulation on NP cells, we stimulated NP cells isolated from degenerative IVDs with 100 ng mL^−1^ GDF6 in culture. After 14 days, GDF6 stimulation significantly increased the expression of the general chondrogenic markers SOX9 (x2.3; *p* < 0.0001), COL2A1 (x4.4; *p* < 0.0001) and ACAN (x9.3; *p* < 0.0001) vs. unstimulated controls (Figure 2A). Moreover, GDF6 stimulation increased the expression of the NP-marker genes KRT8 (x3.7; *p* < 0.0001), KRT18 (x3.2; *p* < 0.0001), KRT19 (x4.2; *p* < 0.0001), FOXF1 (x2.4; *p* < 0.0001), CAXII (x2.6; *p* < 0.0001) and T (x4.4; *p* < 0.0001) vs. unstimulated controls. GDF6 stimulation also significantly increased sulphated GAG production in NP cells in comparison to unstimulated controls (*p* < 0.0001; Figure 2B).

In this system, GDF6 stimulation had no significant effect on the expression of matrix remodelling enzymes known to be important for ECM alterations during IVD degeneration, including MMP3, MMP13, ADAMTS4 and ADAMTS5, in comparison to unstimulated controls (Figure 2C).

### 2.3. Determination of GDF6 Intracellular Signalling and Importance to Healthy NP Phenotype

**Figure 3:**
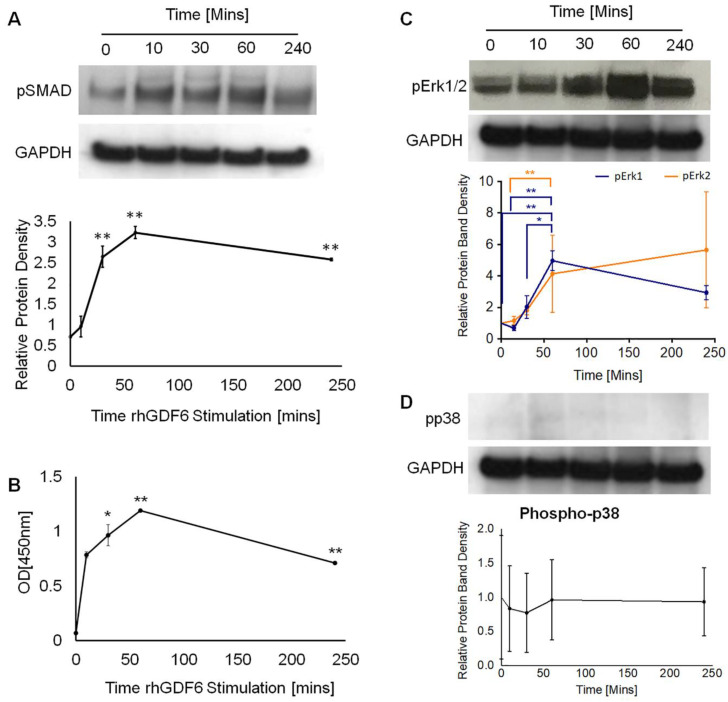
(**A**) Western blot and densitometric analysis of SMAD1/5/8 phosphorylation in NP cells with 100 ng mL^−1^ rhGDF6 stimulation. Data are means *±* SEM (*n* = 3). Statistical significance was determined by Mann–Whitney tests. (**B**) Phospho-Smad1 ELISA analysis of NP cells following 100 ng mL^−1^ rhGDF6 stimulation. Data are means *±* SEM (*n* = 3). Statistical significance was determined by Mann–Whitney tests. (**C**) Western blot and densitometric analysis of ERK1/2 and (**D**) p38 phosphorylation in NP cells following 100 ng mL^−1^ rhGDF6 stimulation. Data are means *±* SEM (*n* = 3). Statistical significance was determined by one-way ANOVA with Tukey correction; * *p* < 0.05, ** *p* < 0.0001.

As we previously reported [34], GDF6 signals through the canonical SMAD1/5/8 pathway. We analysed levels of phospho-SMAD1 in GDF6 stimulated cells through Western blot (Figure 3A) and ELISA assays (Figure 3B) over a 240-min time course. GDF6 stimulation significantly increased SMAD1 phosphorylation at 30 min (*p* = 0.0056), 60 min (*p* = 0.0011) and 240 min (*p* = 0.0046), with peak phosphorylation at 60 min post-stimulation. As other BMP family members have been shown to be able to activate non-SMAD kinase cascades, we investigated whether GDF6 stimulation led to ERK1/2 or p38 MAPK phosphorylation through Western blot analysis (Figure 3C,D). ERK1/2 phosphorylation increased significantly following stimulation from 60 min post-stimulation (*p*-ERK1 0 vs. 60 *p* = 0.0066; 15 vs. 60 *p* = 0.0194; 30 vs. 60 *p* = 0.0136; p-ERK2 0 vs. 60 *p* = 0.0066) and remained elevated after 240 min (Figure 3C). p38 MAPK did not appear to become phosphorylated following GDF6 treatment (Figure 3D).

**Figure 4:**
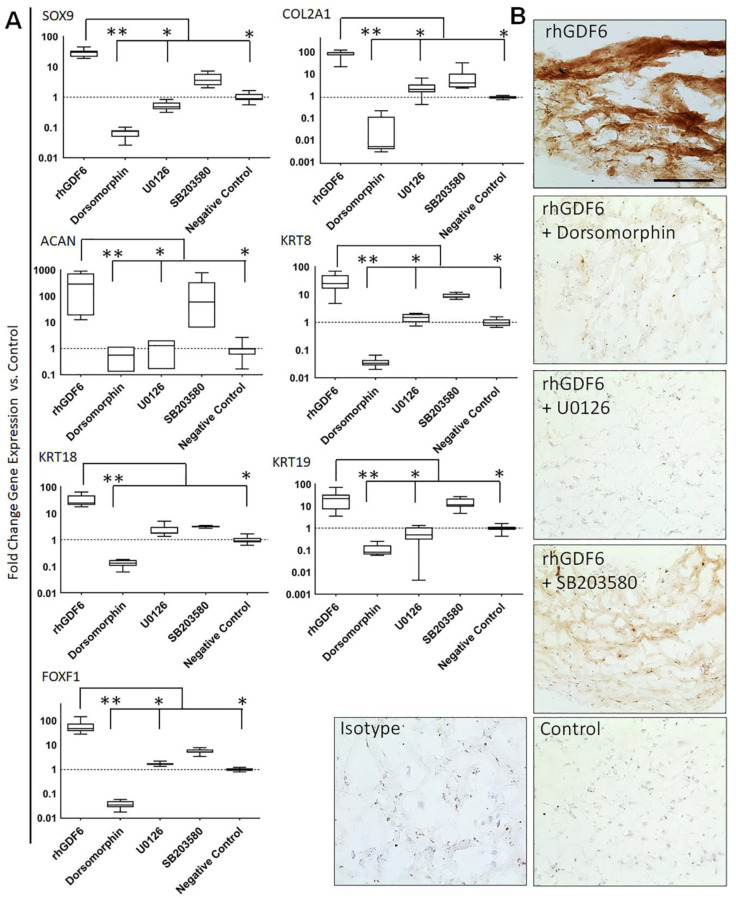
(**A**) QRT-PCR analysis of healthy NP gene expression in NP cells in response to 100 ng mL^−1^ rhGDF6 with and without SMAD1/5/8 (dorsomorphin), ERK1/2 (U0126) and p38 MAPK (SB203580) inhibitors after a 14-day culture. Data are means *±* SEM (*n* = 9). Statistical significance was determined by Kruskal–Wallace multiple comparison tests; ** *p* < 0.0001, * *p* < 0.05. (**B**) Immunohistochemical staining for aggrecan in type I collagen gel NP cultures stimulated with rhGDF6 with and without SMAD1/5/8 or ERK1/2 inhibition. Controls were cultured without rhGDF6, SMAD1/5/8 or ERK1/2 inhibition.

We then aimed to determine the relative effects of these pathways on healthy NP marker gene expression by specifically inhibiting SMAD1/5/8, ERK1/2 and p38 MAPK pathways over 14 days in 3D collagen gel culture and quantifying gene expression through qRT-PCR (Figure 4A). Unblocked cells showed significant upregulations in NP marker gene expression with rhGDF6 stimulation vs. unstimulated controls (*p* < 0.0001). Inhibiting the SMAD1/5/8 pathway led to significant decreases in general chondrogenic genes vs. rhGDF6 stimulated cells (SOX9; *p* < 0.0001), COL2A1; *p* < 0.0001) and ACAN; *p* < 0.0001) and in NP marker gene expression (KRT8; *p* < 0.0001, KRT18; *p* < 0.0001, KRT19; *p* < 0.0001 and FOXF1; *p* < 0.0001), resulting in expression below those of unstimulated controls for all tested genes. Blocking of ERK1/2 also led to attenuation of gene expression, though to a lesser extent than inhibition of SMAD1/5/8 (SOX9; *p* = 0.0002), COL2A1; *p* = 0.05, ACAN; *p* = 0.0082, KRT8; *p* = 0.019, KRT18; *p* = 0.0926, KRT19; *p* = 0.0041 and FOXF1; *p* = 0.0365). In some cases, significant differences in NP gene expression existed between SMAD-inhibited and ERK1/2-inhibited cells (COL2A1; *p* = 0.0273, KRT18; *p* = 0.0132, FOXF1; *p* = 0.036). Inhibiting p38 MAPK signalling had no effect on the rhGDF6-dependent changes in gene expression for any genes assessed, supporting a lack of involvement of p38 MAPK in the rhGDF6 response.

As proteoglycan production is important for healthy NP cell function, we next analysed whether gene expression data correlated with aggrecan protein production in cultures with and without SMAD1/5/8 or ERK1/2 inhibition. rhGDF6 increased aggrecan-positive staining, which was attenuated by inhibition of SMAD1/5/8 and RK1/2 signalling (Figure 4B).

## 3. Discussion

Here, we found that GDF6 receptors were expressed at both the gene and protein level in NP and AF cells extracted from degenerative IVD tissue; however, their expression levels were unaltered by the severity of IVD degeneration. This is important as it suggests that cells within the degenerate IVD environment can still maintain the receptors necessary to respond to GDF6, providing support for exogenous rhGDF6 delivery for treatment of IVD degeneration. We found no significant difference in the level of receptor protein expression between NP and AF cells; however, we observed an increase in BMPR1B gene expression in AF vs. NP cells extracted from discs with a mild grade of degeneration. This suggests that, as with the NP, the AF has the ability to respond to exogenous GDF6, and may indicate that the lack of response to GDF6 reported previously within the AF in vivo may be due to difficulties in delivery of the growth factor within the dense fibrous AF matrix [35] rather than an inability of the AF cells to respond. However, it is worth noting that a study assessing the response of human mesenchymal stem cells (MSCs) and AF cells to silk constructs with and without GDF6 added exogenously, or presented within the silk construct, stimulated matrix gene expression in MSCs but not AF cells. Therefore, it may be necessary to treat the AF region of the degenerate IVDs with an additional growth factor, delivering GDF6 to the NP region only [36]. Further work is required to identify the best solution for AF regeneration.

Delivery of exogenous rhGDF6 within a degenerate IVD would be most easily achieved via injection into the central gelatinous NP region, where there is likely to be a void due to matrix degradation. As such, we wanted to assess the response of human NP cells to rhGDF6. rhGDF6 significantly increased the expression of NP-marker genes (SOX9, ACAN, COL2A1, KRT8, KRT18, KRT19, FOXF1, CA12 and T) in NP cells and sGAG production following 14 days in culture. These results are similar to those observed for rhGDF6 stimulation of human MSCs [16,37] and provide further evidence of an anabolic role for GDF6 in healthy IVD homeostasis. rhGDF6 stimulation had no effect on the expression of catabolic enzymes (MMPs, ADAMTSs) in comparison to unstimulated controls, although levels were not induced in the system (e.g., through stimulation with a catabolic cytokine, such as IL-1β), hence GDF6 may not be able to reduce expression below basal levels and further work is required to interrogate this aspect in more detail.

Taken together, the above suggests that IVD cells remain able to respond to GDF6 presented to their cell surface, even in severely degenerated tissues, and that GDF6 stimulation has the potential to increase anabolic gene expression and sGAG production while not influencing the expression of matrix remodelling enzymes. The change in cellular phenotype observed during IVD degeneration suggests a lack of response by degenerative NP cells in vivo to endogenous GDF6. This may indicate that during degeneration, there is either reduced expression of GDF6 and, therefore, receptor binding, or an alteration to the relationship between receptor–ligand binding and the adoption of GDF6-effector gene expression, potentially involving other biological cues. In the former case, the supplementation of exogenous GDF6 to the degenerate disc may redress this homeostatic balance. In the latter, a deeper understanding of intra-cellular GDF6 signalling is required for effective therapeutic use.

Therefore, we aimed to determine downstream signalling pathways activated by GDF6 signalling. As expected for a BMP family member, rhGDF6 stimulation phosphorylated SMAD1, indicating activation of the SMAD1/5/8 pathway. Specific inhibition of SMAD1/5/8 resulted in significant attenuation of NP gene expression. rhGDF6 stimulation was also found to activate ERK1/2 signalling in NP cells. ERK1/2 inhibition decreased healthy NP-marker gene expression on rhGDF6 stimulation, returning expression to baseline levels. p38 MAPK inhibition did not consistently downregulate NP maker genes. More importantly, p38 MAPK inhibition did not influence ACAN expression at either mRNA or protein level. This result is consistent with previous work investigating GDF5 signalling, where SMAD1/5/8 and ERK1/2 was phosphorylated but not p38 MAPK [38], and with our work on adipose-derived stem cells, which similarly showed signalling via SMAD1/5/8 and ERK1/2 but not p38 MAPK [34].

The degenerative IVD is a proinflammatory environment with notable increases in IL-1β and TNFα, which are known to accelerate the development of degenerative phenotypes and catabolic gene expression profiles [32,39,40]. This proinflammatory environment could have a significant impact on GDF6 signalling. Current evidence suggests that IL-1β may downregulate GDF family member expression in disc cells [41], although the mechanism for this remains to be fully resolved. One possible proposed mechanism is via miRNA-mediated inhibition, with the recent identification of GDF5 as a target of IL-1β inducible miRNAs (e.g., miR-7 [42]), significantly reducing GDF5 mRNA expression.

Alternatively, a direct interaction between proinflammatory signalling and GDF6 signalling may be responsible for decreased anabolic gene expression in cells still able to respond to GDF6. For example, the presented data demonstrate that in addition to SMAD1/5/8 signalling, rhGDF6 is able to activate ERK1/2, which is known to interact with IL-1 β signalling. Having shown here that these pathways are critical to rhGDF6-mediated upregulations in NP-marker gene expression, and that GDF6 receptors remain present through the degeneration process, it follows that this convergence of signalling may have a role in attenuating or altering cellular responses to GDF6. Indeed, a recent publication has provided evidence of contrasting cellular responses to BMP family signalling and IL-1β/TNFα signalling through ERK1/2 and p38, despite both converging on the transcription factor RUNX2 [43]. Although in the IVD, and for GDF6, this relationship requires further investigation, this framework provides an interesting perspective that may be important for optimisation of GDF6-based therapeutics in IVD degeneration. It may be interesting to evaluate whether rhGDF6 treatment with an anti-inflammatory molecule, e.g., IL-1 receptor antagonist, in order to fully neutralise the negative effects of the degenerate niche and repair/restore tissue function may be of additional benefit.

## 4. Materials and Methods

### 4.1. Extraction of NP and AF Cells

Human IVD tissue was obtained at surgical intervention in patients (*N* = 27) with chronic low back pain following ethical approval and informed consent of patients (Supplementary Table S1). A section of each tissue sample was processed to paraffin wax for grading according to a published 12-point histological scale [33]. To extract cells, removed disc tissue was dissected into NP and AF. The tissue was finely minced and digested with 300 U mL^−1^ pronase (Calbiochem) in Dulbecco’s Modified Eagle’s Medium (DMEM) for 30 min at 37 °C and subsequently washed twice in DMEM. NP and AF cells were isolated at 37 °C in 0.25% collagenase type II (Gibco)/0.1% hyaluronidase (Sigma-Aldrich, Gillingham, UK) in serum-free media containing antibiotics. The digested cell suspension was filtered through a 40 µm cell strainer (BD Falcon) to remove tissue debris. Cells were centrifuged at 400 g for 5 min and seeded into tissue culture flasks. Cells were expanded in DMEM containing 10% foetal bovine serum (FBS), 110 mg L^−1^ sodium pyruvate, 100 U mL^−1^ penicillin, 100 µg mL^−1^ streptomycin and 0.25 µg mL^−1^ amphotericin, hereafter referred to as expansion media.

### 4.2. RNA Extraction and Quantitative Real-Time PCR

Cells were disrupted in TRI Reagent (Sigma-Aldrich) and RNA was isolated, quantified through UV spectroscopy (NanoDrop, Thermo Scientific, Cambridge, UK) and cDNA synthesised. To analyse the gene expression profiles of different NP and AF cDNA samples, qPCR reactions were undertaken using either the TaqMan or SYBR Green qPCR systems for BMPR1A, BMPR1B, BMPR2, GDF6, SOX9, ACAN, COL2A1, KRT8, KRT18, KRT19, FOXF1, CA12, T, MMP3, MMP13, TIMP1, ADAMTS4, ADAMTS5, EIF2B1 and MRPL19. FAM-BHQ1 probes were utilised at a concentration of 450 mM and optimal primer concentration was determined empirically. Predesigned primers (PrimerDesign, Eastleigh, UK) and premixed primer and probe TaqMan assays (Life Technologies, Paisley, UK), were used as per the manufacturers’ instructions. QRT-PCR reactions were run on an Applied Biosystems StepOne Plus Real Time PCR System. The reaction mastermix contained 5 μL 2 × Lumino Ct qPCR Readymix, 1 μL forward primer, 1 μL reverse primer and 0.5 μL probe and 0.5 μL 40 × ROX internal reference dye. For the predesigned primers, and the predesigned primers and probe sets, 1 μL of the predesigned mix was added per reaction and the volume of water increased to ensure 8 μL volume of mastermix (including 5 μL of SYBR green or Taqman mastermix) per reaction. The mastermix was vortexed and 8 μL pipetted into each well of the 96-well plate. Biological samples were examined in triplicate and 2 μL of cDNA was pipetted into each well. A positive and negative sample was run for each gene examined to ensure no false positives; total human RNA and molecular grade H_2_O replaced cDNA, respectively. The reactions were cycled under the following conditions: 95 °C for 20 s, followed by 40 cycles of 95 °C for 1 s and 60 °C for 20 s. Optimised primer concentration and primer/probe sequences for the genes utilised throughout this study are detailed in Supplementary Table S2. Data were analysed according to the 2^-ΔCt^ method outlined by Livak and Schmittgen [44] and normalised to either MRPL19 (Figure 1 and Figure 2), or both MRPL19 and EIF2B1 (Figure 4).

### 4.3. GDF Receptor Protein Profiles

Protein was extracted from patient-matched NP and AF cells in culture (passage 1) using radioimmunoprecipitation assay (RIPA) buffer (50 mM Tris, pH 8.0, 150 mM NaCl, 0.1% SDS, 5 nM EDTA, 0.5% (w/v) sodium deoxycholate and 1% Nonidet P-40) supplemented with protease and phosphatase inhibitors as per the manufacturer’s instructions (Thermo Scientific) at 4 °C. Insoluble material was removed from cell lysates by centrifugation (10,000× *g*/10 min/4 °C) and the remaining protein concentration of the supernatant quantified. For Western blot analysis, 20 µg total cell lysate was loaded into the wells of 4–12% Bis/Tris Bolt gels (Life Technologies, Paisley, UK) and separated by SDS-PAGE. After electrophoresis, proteins were transferred to polyvinylidene fluoride (PVDF) membranes (Thermo Fisher Scientific, Altrincham, UK) and incubated with blocking buffer. Subsequently, membranes were incubated with primary antibodies (Supplementary Table S3) diluted in blocking buffer containing 0.1% (v/v) Tween 20 (Sigma) overnight at 4 °C. Membranes were washed 5 times in Tris Buffered Saline with 0.1% (v/v) Tween 20 (TBST). Relevant horseradish peroxidase (HRP)-conjugated secondary antibodies were incubated with membranes for 1 h at room temperature. Following incubation, the membranes were washed 5 times with TBST and developed using ECL chemiluminescent reagent (PERKinElmer, Beaconsfield, UK) according to the manufacturer’s instructions and exposed to photographic film. The density of each protein band was quantified using the Syngene imaging system, and the ratio of the density of bands to the density of GAPDH protein bands calculated.

### 4.4. ELISA and Western Blot Analysis of rhGDF6 Signalling Pathway Activation

The activation of SMAD and non-SMAD pathways by rhGDF6 stimulation in culture was investigated. Cells were serum-starved for 24 h prior to all experiments. Subsequently, starvation media were replaced with serum-free media containing 100 ng mL^−1^ rhGDF6 (previously optimised in [16]) (PeproTech cat no. 120-04). At defined intervals, GDF6 media were removed, cells were washed twice with ice-cold PBS and protein was extracted in RIPA buffer or ELISA Lysis Buffer (RayBiotech, Peachtree Corners, GA, USA). To determine SMAD1 phosphorylation, a phospho-SMAD1 ELISA (RayBiotech) was performed according to the manufacturer’s instructions. Cells were seeded into wells at a density of 5 × 10^4^ cells per cm^2^, and after serum starvation, cells were washed with PBS and incubated in serum-free NP expansion media with and without rhGDF6. After incubation, cells were washed three times with ice-cold PBS and protein was extracted with cell lysis buffer (provided with ELISA assay) supplemented with protease and phosphatase inhibitors (Life Technologies). Protein was quantified through Bicinchonic Acid (BCA) assay (Pierce) and normalised. Phospho-SMAD1 ELISA assays (RayBiotech) were performed according to the manufacturer’s instructions and Smad1 phosphorylation was quantified through measuring absorbance at 450 nm.

Activation of ERK1/2 and p38 MAPK pathways by rhGDF6 stimulation was investigated through Western blot using antibodies against phosphorylated forms of ERK1/2 and p38 MAPK. Protein extraction and Western blots were performed as above, and membranes were probed with phospho- and pan-ERK1/2 and p38 MAPK antibodies (Cell Signaling Technology, Leiden, The Netherlands). Primary antibodies used in this study are in Supplementary Table S3.

### 4.5. Pathway-Specific Inhibition in 3D Collagen Gel Culture

To determine the relative effects of specific pathways on NP-marker gene expression, NP cells were cultured in a 3D type I collagen gel culture for two weeks with and without additional rhGDF6 stimulation at 100 ng mL^−1^. NP cells were loaded at high density (1.0 × 10^6^ cells mL^−1^) into type I collagen gels (Devro) (3 mg mL^−1^) and 100 µL placed into 0.4-µm high-density cell culture inserts in 24-well plates. After the gels were set, they were stabilised for 24 h in NP expansion media, after which the media were changed for NP-differentiation media (high-glucose DMEM, 1% FCS, insulin-transferrin-selenium (ITS-X), 100 µM ascorbic acid-2-phosphate, 1.25 mg mL^−1^ bovine serum albumin, 10^−7^ M dexamethasone, 5.4 µg mL^−1^ linoleic acid, 40 µg mL^−1^ L-proline, 100 U mL^−1^ penicillin, 100 µg mL^−1^ streptomycin and 0.25 µg mL^−1^). To investigate the relative role of different rhGDF6-activated pathways in NP cell phenotypes, SMAD1/5/8, ERK1/2 and p38 MAPK were selectively blocked using the small molecule inhibitors dorsomorphin (10 µM), U0126 (10 µM) and SB20358 (10 µM), respectively. Media were changed every 48 h. At each media change, and at the start of culture, cells were pre-blocked with GDF6-free media containing the relevant inhibitor for 1 h prior to the addition of GDF6-inhibitor containing media. After two weeks of culture, RNA was extracted from the gels and qRT-PCR analysis of NP-marker gene expression was performed.

### 4.6. Immunohistochemical Staining of NP Cell Seeded Type I Collagen Gels

Collagen constructs were embedded in optimum cutting temperature (OCT) compound, snap-frozen in liquid nitrogen and cryosectioned into 6 μm sections. Aggrecan production was investigated through immunohistochemical (IHC) analysis of gels. Briefly, gels were embedded into OCT compound and frozen in liquid nitrogen. Gels were cut into 8 µm thick cross-sections and collected onto SuperFrost Plus slides. For IHC analysis, the sections were allowed to equilibrate to room temperature, formalin-fixed and blocked prior to addition of the aggrecan primary antibodies [45] and incubated at 4 °C overnight. The relevant HRP-conjugated secondary antibody was added after washing (5 × 5 min TBS with 0.1% (v/v) Tween 20; TBST) and incubated at room temperature for 1 h. Following washing with TBST, specific staining was visualised through incubation at room temperature with DAB chromagen. Bright field images were acquired from three independent gel cultures and representative images depicted.

### 4.7. Statistical Analysis

Statistical significance in qRT-PCR analysis of GDF6 receptors in NP and AF cells across a range of grades was determined by Brown–Forsythe and Welch ANOVA tests. Comparisons between NP and AF cells of the same grading were made using Mann–Whitney tests. To statistically compare GDF6 receptor expression in AF and NP cells through densitometric analysis of Western blots, Mann–Whitney tests were performed. Statistical significance in qRT-PCR analysis of healthy NP gene expression and the effects of Smad and ERK1/2 inhibition was assessed through unpaired T-Tests with Welch’s correction and Kruskal–Wallace multiple comparison tests, respectively. Differences in sulphated GAG production were statically compared through unpaired T-Tests with Welch’s correction. Phospho-Smad1 ELISA, Smad1/5/8 was analysed using Mann–Whitney tests and ERK1/2 and p38 phosphorylation was analysed by one-way ANOVA with Tukey correction. For all analyses, a value of *p* < 0.05 was considered to be statistically significant. Values are reported as means ± SEM unless otherwise stated. Statistical analysis was performed using GraphPad Prism software (v8.0.0; GraphPad Software Inc., San Diego, CA, USA).

## 5. Conclusions

In conclusion, we show here that NP and AF cells express GDF6 receptors across a range of degeneration grades and that rhGDF6 stimulation of NP cells significantly increased healthy NP marker gene expression and sGAG production, with SMAD1/5/8 and ERK1/2 pathway activation required for this effect. In summary, NP and AF cells in degenerate discs remain responsive to GDF6 signalling and targeting downstream SMAD and ERK1/2 activation may further enhance the efficacy of GDF6 therapeutics.

## Supplementary Materials

Supplementary materials can be found at https://www.mdpi.com/1422-0067/21/19/7143/s1.
