# Supplementary material for: Regenerative Response of Degenerate Human Nucleus Pulposus Cells to GDF6 Stimulation
Source: Int J Mol Sci. 2020 Sep 27;21(19):7143. doi: 10.3390/ijms21197143 (PMC7582366; doi:10.3390/ijms21197143)

Regenerative response of degenerate human nucleus pulposus cells to GDF6 stimulation

Tom Hodgkinson[1], Hamish T J Gilbert[1], Tej Pandya[1], Ashish Diwan[2], Judith A. Hoyland[1,3] and Stephen M. Richardson[1*]

[1] Division of Cell Matrix Biology and Regenerative Medicine, School of Biological Sciences, Faculty of Biology, Medicine and Health, University of Manchester, Oxford Road, Manchester M13 9PT, UK.

[2] St George & Sutherland Clinical School, University of New South Wales, Sydney, Australia.

[3] NIHR Manchester Biomedical Research Centre, Central Manchester Foundation Trust, Manchester Academic Health Science Centre, Manchester, United Kingdom.

Original uncropped western blots

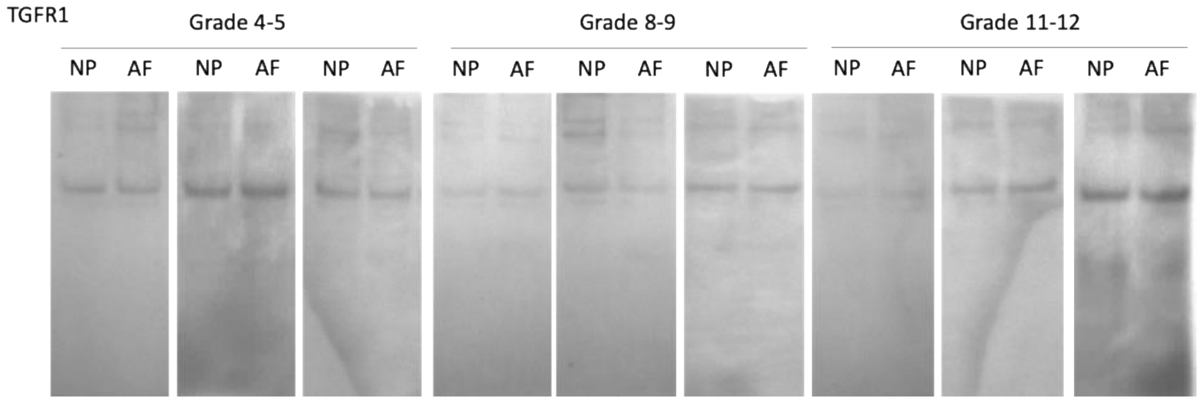

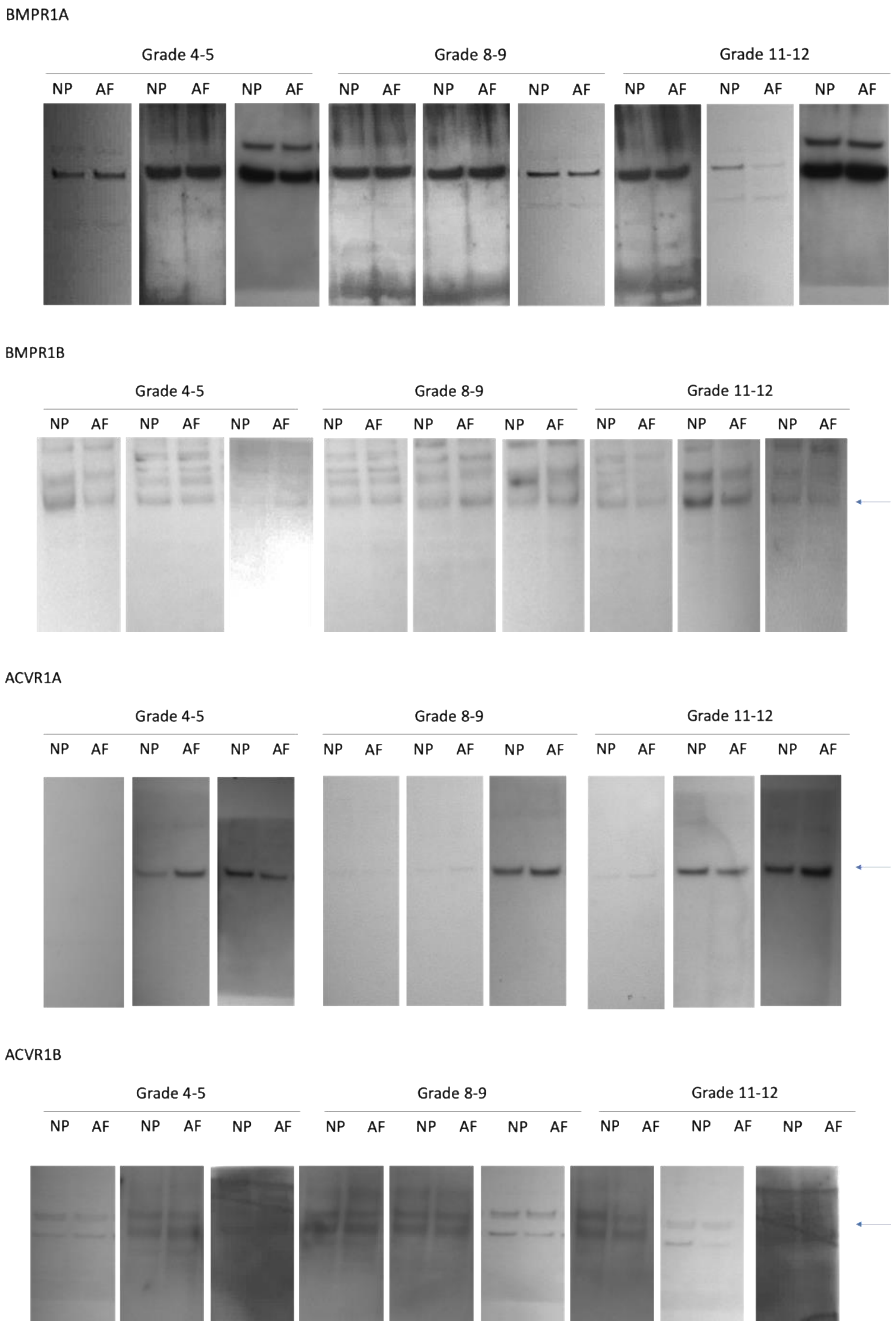
BMPR1A
Grade 4-5
Grade 8-9
Grade 11-12
NP AF NP AF NP AF NP AF NP AF NP AF NP AF NP AF NP AF
BMPR1B
Grade 4-5
Grade 8-9
Grade 11-12
NP AF NP AF NP AF NP AF NP AF NP AF NP AF NP AF NP AF
ACVR1A
Grade 4-5
Grade 8-9
Grade 11-12
NP AF NP AF NP AF NP AF NP AF NP AF NP AF NP AF NP AF
ACVR1B
Grade 4-5
Grade 8-9
Grade 11-12
NP AF NP AF NP AF NP AF NP AF NP AF NP AF NP AF NP AF

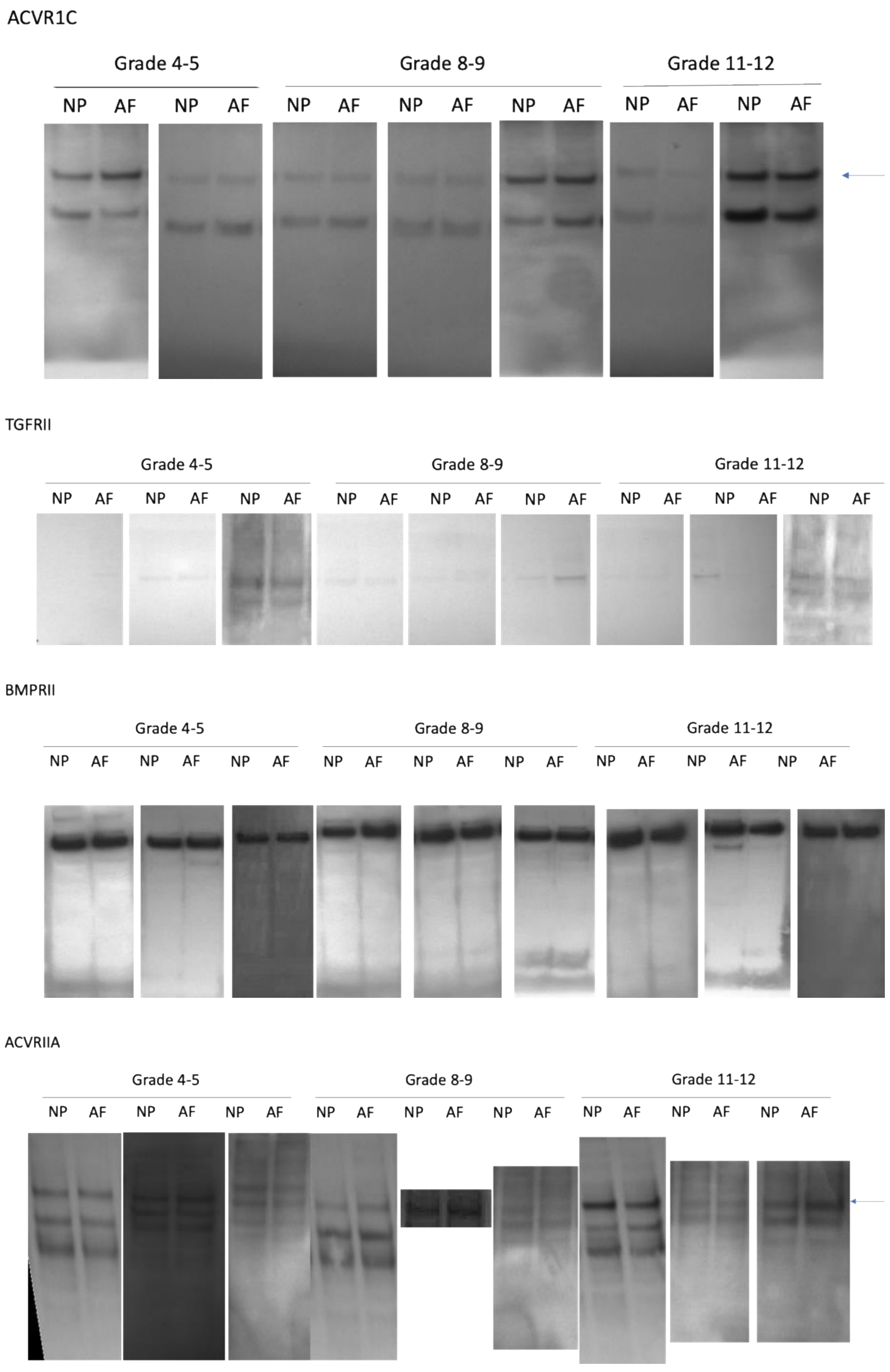
ACVR1C
Grade 4-5
Grade 8-9
Grade 11-12
NP AF NP AF NP AF NP AF NP AF NP AF NP AF
TGFRII
Grade 4-5
Grade 8-9
Grade 11-12
NP AF NP AF NP AF NP AF NP AF NP AF NP AF NP AF NP AF
BMPRII
Grade 4-5
Grade 8-9
Grade 11-12
NP AF NP AF NP AF NP AF NP AF NP AF NP AF NP AF NP AF
ACVRIIA
Grade 4-5
Grade 8-9
Grade 11-12
NP AF NP AF NP AF NP AF NP AF NP AF NP AF NP AF NP AF

ACVRIIB

Grade 4-5 Grade 8-9 Grade 11-12

NP AF NP AF NP AF NP AF NP AF NP AF NP AF

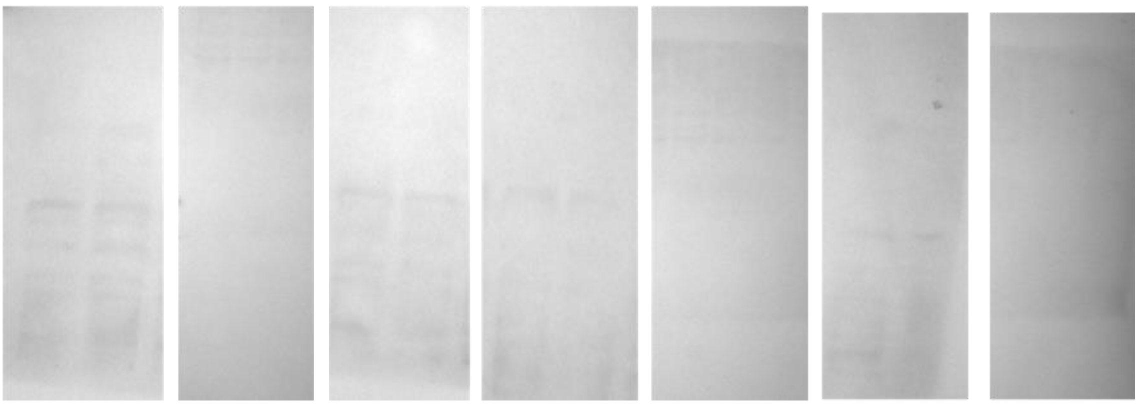

GAPDH

Grade 4-5 Grade 8-9 Grade 11-12

NP AF NP AF NP AF NP AF NP AF NP AF NP AF NP AF NP AF

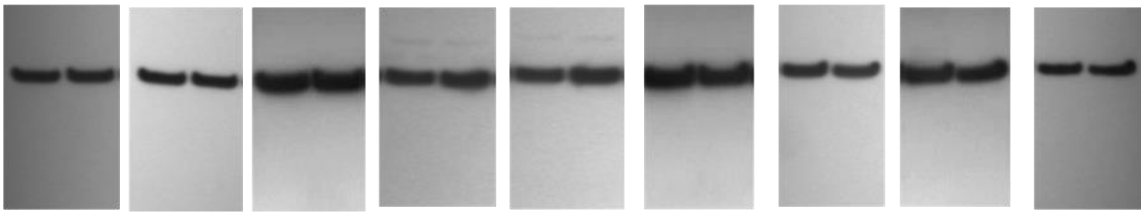

pSMAD

T0 T10 T30 T60 T240. T0 T10. T30 T60 T240. T0 T10 T30. T60 T240

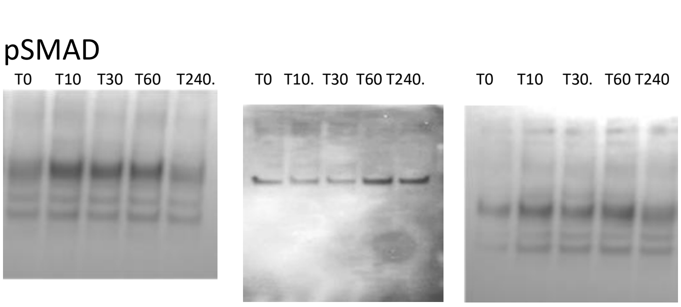

p-ERK

T0 T10 T30 T60 T240. T0 T10 T30 T60 T240. T10 T30 T60 T240 T10 T30 T60 T240

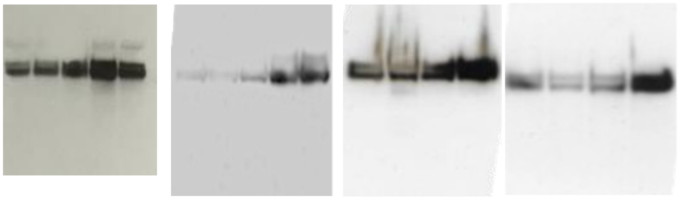

pp38

T0 T10 T30 T60 T240. T0 T10. T30 T60 T240. T0 T10 T30. T60 T240

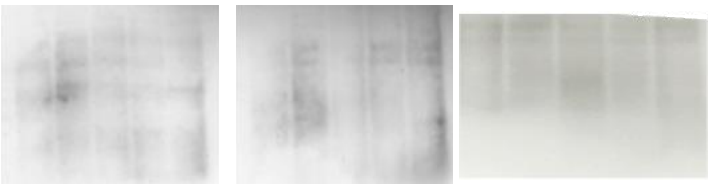

GAPDH

T0 T10 T30 T60 T240. T0 T10. T30 T60 T240. T0 T10 T30 T60 T240. T0 T10 T30 T60 T240

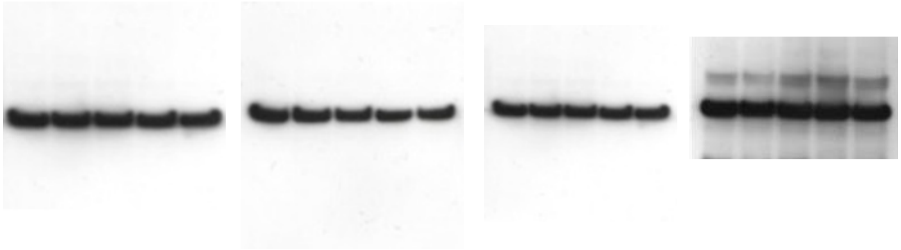

Supplement: Supplementary file 1 [file ijms-21-07143-s001.pdf]
